# Evaluation of Antioxidant Activities of Aqueous Extracts and Fractionation of Different Parts of *Elsholtzia ciliata*

**DOI:** 10.3390/molecules17055430

**Published:** 2012-05-09

**Authors:** Xiangping Liu, Jia Jia, Lei Yang, Fengjian Yang, Hongshuang Ge, Chunjian Zhao, Lin Zhang, Yuangang Zu

**Affiliations:** 1 State Engineering Laboratory for Bioresource Eco-Utilization, Northeast Forestry University, Harbin 150040, China; Email: lxp110@hotmail.com (X.L.); yangfj@nefu.edu.cn (F.Y.); swx05131ghs@126.com (H.G.); zcjsj@163.com (C.Z.); zhanglin6600@sina.com (L.Z.); 2 College of Animal Science & Veterinary Medicine, Heilongjiang Bayi Agricultural University, Daqing 163319, China; 3 Preclinical Medicine Department of Daqing Medical College, Daqing 163319, China; Email: jiajiaplay2006@163.com (J.J.)

**Keywords:** *Elsholtzia ciliata*, free-radical scavenging activity, antioxidant activity, phenolics content

## Abstract

The aim of this study was to investigate the antioxidant and free-radical scavenging activity of extract and fractions from various parts of *Elsholtzia ciliata*. The inflorescences, leaves, stems and roots of *E. ciliata* were extracted separately and two phenolic component enrichment methods: ethyl acetate-water liquid-liquid extraction and macroporous resin adsorption-desorption, were adopted in this study. The antioxidant activities of water extracts and fractions of *E. ciliata* were examined using different assay model systems *in vitro*. The fraction root E (purified by HPD300 macroporous resin) exhibited the highest total phenolics content (497.2 ± 24.9 mg GAE/g), accompanied with the highest antioxidant activity against various antioxidant systems *in vitro* compared to other fractions. On the basis of the results obtained, *E. ciliata* extracts can be used potentially as a ready accessible and valuable bioactive source of natural antioxidants.

## 1. Introduction

The common free radicals are oxygen-reactive species (ROS), which are formed during natural metabolism and are in dynamic balance between their biosynthesis and their removal by antioxidant systems in human bodies. When the mechanism of antioxidant protection becomes unbalanced by factors such as ageing, deterioration of physiological functions may occur, resulting in oxidative stress and thus can lead to cell injury and various relevant diseases such as cardiovascular diseases, cancer and accelerated ageing [1,2,3]. However, the innate defense systems of body may be supported by antioxidative compounds taken as foods, cosmetics and medicine. Although the most widely used antioxidants are synthetic antioxidants, such as butylated hydroxytoluene (BHT) and butylated hydroxyanisole (BHA), their toxic properties and unwanted side effects limit their widespread use [4,5,6,7]. Hence, the development of alternative antioxidants from natural sources has considerable prospects.

It is common knowledge that naturally occurring substances in higher plants have antioxidant activity. In the recent years, more attention has been paid to the protective biochemical functions of naturally occurring antioxidants in the cells of the organisms containing them [8,9,10,11,12,13].

There has been a growing interest in Elsholtzia species because of its long history for over thousands of years. The studies of Elsholtzia species are mainly focused in constituents of essential oil [14,15,16], antivirus function [17] and so on. *Elsholtzia ciliata*, a plant which is also known as “Xiang Ru” in China, is an annual herb that belongs to the Elsholtzia species that is widely distributed throughout China, Korea and Europe. The seeds of *E. ciliata* are powdered and used for flavoring foods. The chemical components of the crude drug are essential oil, elsholtzia ketone, flavonoids, steroids [18] and triterpenes. As a favorite and widely used medical herb in traditional Chinese medicine (TCM), the herb has been used for the treatment of fever, headache, diarrhea and edema [19]. Moreover, *E. ciliata* has been officially listed in the menu of edible herbs by the Ministry of Health of China.

Researchers have sought to isolate powerful and nontoxic natural antioxidants from edible plants not only to prevent autoxidation and lipid peroxidation, but recently also to replace synthetic antioxidants. *E. ciliata* has been more often used as a food than for medicinal purposes. To our knowledge, there is no report in the literature on the antioxidant potential of the whole plant. The main objectives of this work were to determine the phenolics content, evaluate the antioxidant activities of aqueous extracts and fractionation from different parts of *E. ciliata* by two different enrichment methods: ethyl acetate-water liquid-liquid extraction and macroporous resin adsorption-desorption, were adopted.

## 2. Results and Discussion

### 2.1. Extraction Yield and Total Phenolics Content of Different Parts of *E. ciliata* Plant

**Figure 1:**
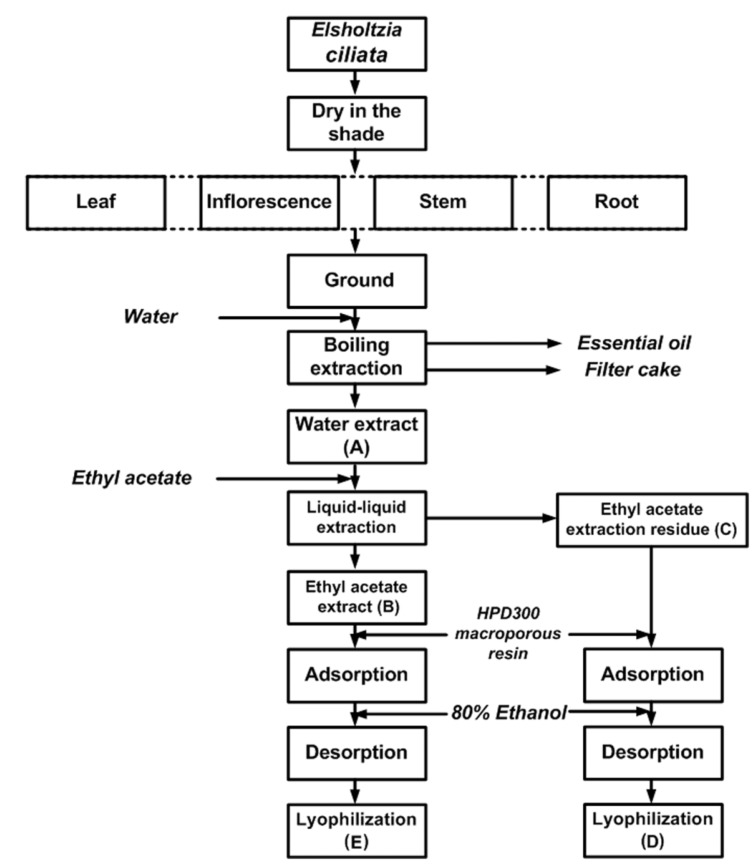
Isolation procedure for extractsof *E. ciliata* plant parts.

**Figure 2:**
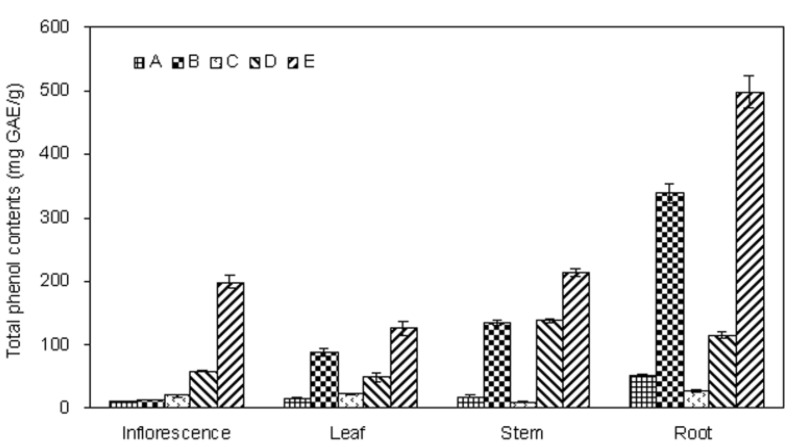
Total phenolics in fractions of different parts of *E. ciliate*.

The amount of materials that can be extracted from a plant depends on the vigour of the extraction procedure and the possibility also exists of sample-to-sample variation in the extracted material. The isolation procedure for extracts of *E. ciliata* plant is shown in Figure 1, the results on the yields of extraction are summarized in Table 1, and the total phenolics contents of *E. ciliata* plant parts are shown in Figure 2. The crude water extract yield of all plant parts varied from 30.64 ± 1.04 to 5.60 ± 0.16 g/100 g dry raw materials. Among all the plant parts, the crude water extract of leaf obtained the highest extraction yield (30.64 ± 1.04 g), while the root yielded the lowest (5.60 ± 0.16 g). The yield of all the fractions is presented in different order. 

**Table 1:** Yields of extraction of *E. ciliata* in different plant parts.

Yield	Leaf (g)	Inflorescence (g)	Stem (g)	Root (g)
A ^a^	30.64 ± 1.04	15.26 ± 0.34	10.56 ± 0.43	5.60 ± 0.16
B ^b^	2.38 ± 0.09	2.49 ± 0.09	2.49 ± 0.09	1.41 ± 0.05
C ^b^	2.39 ± 0.07	2.28 ± 0.09	2.29 ± 0.06	3.49 ± 0.09
D ^c^	0.13 ± 0.01	0.09 ± 0.01	0.12 ± 0.01	0.29 ± 0.01
E ^c^	0.54 ± 0.02	0.48 ± 0.01	0.65 ± 0.02	0.20 ± 0.01

^a^ From 100 g dry raw materials; ^b^ From 5 g A; ^c^ From 1 g B or C; A, B, C, D and E, the same as Figure 1.

**Figure 3:**
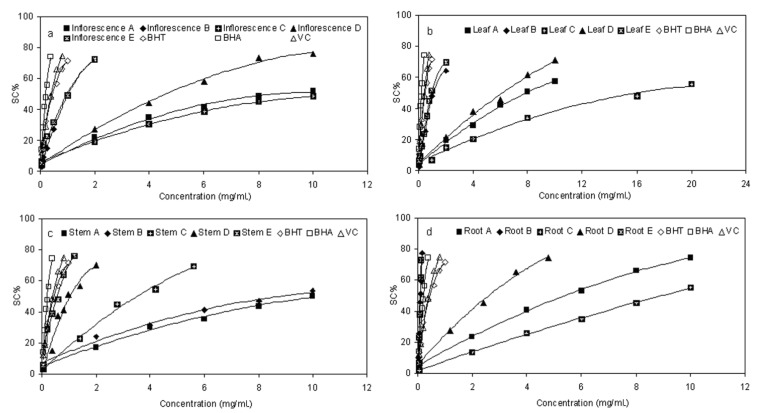
DPPH radical scavenging activity of sequential extracts in different parts of *E. ciliata*. Each value is the mean of triplicate measurements; Concentrations relative to Scavenging activity(SC%); Regression analysis formulas were listed under each graph.

Many studies have pointed out that there is a positive correlation between antioxidant activity potential and amount of phenolic compounds of the extracts [20,21,22]. Therefore, the amount of total phenolics in the extracts was determined in this study. The total phenolics content of the *E. ciliata* extracts was determined through a linear gallic acid standard curve (y = 0.0039x − 0.009; R^2^ = 0.9994) and expressed as milligram of gallic acid equivalents per gram of extract (mg GAE/g). As can be seen from Figure 3, the total phenolics content was varying in different extracts of *E. ciliata* plant. The amount of the total phenolics was significantly higher in root E (497.2 ± 24.9 mg GAE/g) compared with the other parts of plant, and followed by stem E (213.1 ± 6.2 mg GAE/g), inflorescence E (198.2 ± 10.1 mg GAE/g). Fraction E contained the highest phenolics content among all five fractions. Furthermore, fraction B was found to be the second one, followed by fraction E. This result suggested that the phenolic compounds might selectively be extracted into the ethyl acetate fraction from *E. ciliata* and be further enriched by macroporous resin technology, but suitable adjustment of the extraction parameters of process is required. 

### 2.2. Scavenging Effect on DPPH Radical

The ability of fractions from *E. ciliata* to quench reactive species by hydrogen donation was measured through the DPPH radical scavenging activity assay [23,24]. As a kind of stable free radical, DPPH can accept an electron or hydrogen radical to become a stable diamagnetic molecule, which is widely used to investigate radical scavenging activity [25]. The analysis of DPPH scavenging activity (SC%) are presented in Figure 3, and the EC_50_ was calculated by the regression analysis formulas under graph. We observed that, different distracts in different parts of *E. ciliata* plant showed a concentration-dependent DPPH radical scavenging activity. The five fractions at each sequential step exhibited varying degrees of scavenging capacities. On the whole, the activity of the fractions towards the DPPH radical increased in the order C < A < D < B < E. Among the five fractions, fraction E exhibited the highest scavenging activity with the lowest EC_50_ value of 0.97, 0.57, 1.03, 0.09 mg/mL in leaf, stem, inflorescence and root, respectively, which is comparable and even lower to that of BHT (0.45), BHA (0.21), and Vc (0.41). Especially the root fractionE showed significantly stronger scavenging potency than that of other parts of plant, which is followed by fraction B. This result suggested that some components within ethyl acetate fractions were strong radical-scavenging components. On the other hand, fractions E and D were superior to fractions B and D, respectively, which proved that elution process of HPD300 macroporous resin by 80% ethanol also has a remarkable effect to concentrate radical-scavenging components.

### 2.3. Reducing Power

**Figure 4:**
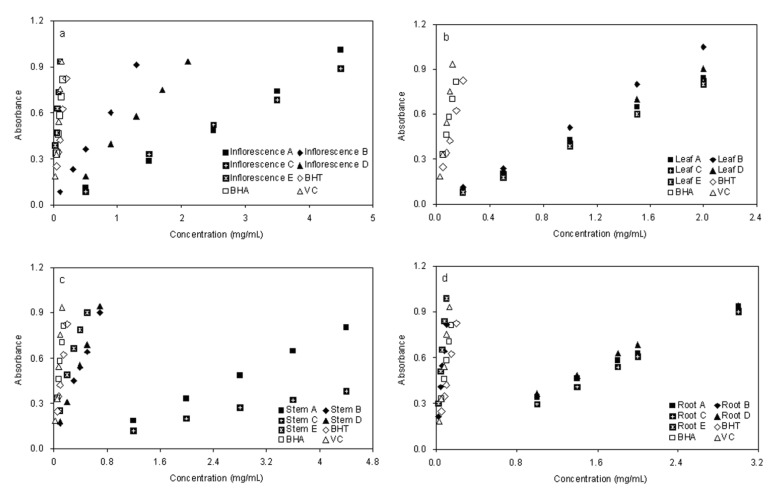
Reducing Power for Fractions of Different Parts of *E. ciliata*. Concentrations Relative to Absorbance and Each Value are the Mean of Triplicate Measurements.

The potassium ferricyanide reduction method was used to evaluate the reducing power of plant phenolics, which is a widely used method in measuring antioxidant activity of phenolic compounds. In this assay, the antioxidants present in the test solution can reduce the Fe^3+^/ferricyanide complex to the ferrous form by donating an electron. Increasing absorbance of the reaction mixture at 700 nm indicates an increase in the reducing power [26]. In the present study, as shown in Figure 4, the reducing power of the five fractions exhibited a similar concentration-dependent activity pattern as given in the DPPH analysis. Among all fractions, inflorescence fraction E (0.937 ± 0.024 at 0.1 mg/mL) (Figure 4a), root B (0.817 ± 0.027 at 0.1 mg/mL) and root E (0.987 ± 0.019 at 0.1 mg/mL) (Figure 4d) were found to be superior to the other fractions, but significantly lower than the positive control BHT (0.423 ± 0.011 at 0.1 mg/mL), BHA (0.580 ± 0.015 at 0.1 mg/mL) and comparable with Vc (0.754 ± 0.011 at 0.1 mg/mL). Fractions C and A possessed weaker activity in all four parts for this test system. Meanwhile, fractions E were the strongest radical reducer, compared with the others. However, there were differences in the results among fractions D of different parts of plant. This is comparable with fractions A and C for leaf and root, which are different The main objectives of this work were to determine the phenolics content, evaluate the antioxidant activities of aqueous extracts and fractionation from different parts of *E. ciliata* by two different enrichment methods, ethyl acetate–water liquid liquid extraction and macroporous resin adsorption – desorption, were adopted.

### 2.4. Ferric-reducing Antioxidant Power (FRAP)

**Figure 5:**
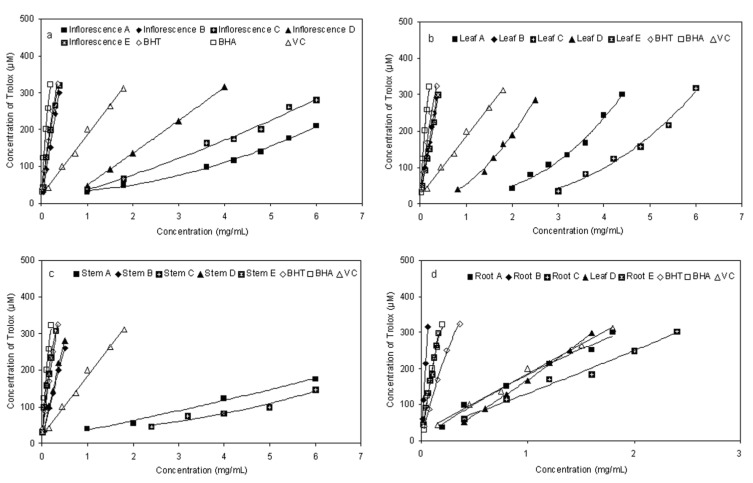
Ferric-reducing antioxidant power (FRAP) assay for fractions of different parts of *E. ciliata*.

FRAP is a simple direct test for measuring antioxidant activity by quenching ferric reducingcapacity [8], which was used in many studies as a general method [27,28,29]. In this assay, extracts were used in a redox-linked reaction whereby the antioxidants presented in the sample act as the oxidants. Reduction of the ferric-tripyridyltriazine to the ferrous complex forms an intense blue colour which can be measured at a wavelength of 593 nm. The intensity of the colour is related to the amount of antioxidant reductants in the extracts. The trend for ferric ion-reducing activities of different fractions from *E. ciliata* in the present study is shown in Figure 5. The absorbance clearly increased due to the formation of the Fe^2+^-TPTZ complex with increasing concentration. The higher reducing activity was for the fraction B and E, compared to those of the other fractions (Figure 5). Similar to the results obtained from the DPPH and reducing power assay, the fraction D showed relatively strong ferric ion-reducing activity, while the fractions A and C showed lower ferric ion-reducing activities.

## 3. Experimental Section

### 3.1. Plant Materials

The plant *E. ciliata* (Labiatae) was collected from the Botanical Garden of Northeast Forestry University and authenticated by Prof. Xiaoying Yuan from the State Engineering Laboratory for Bioresources Eco-Utilization, Northeast Forestry University, China. A specimen was deposited in the Herbarium of this Key Laboratory. The plant samples, including inflorescence, leaf, stem and root, were washed and dried in the shade at room temperature. The dried plants were powdered using a disintegrator.

### 3.2. Chemicals

Folin-Ciocalteu’s reagent, 1,1-diphenyl-2-picrylhydrazyl (DPPH), 2,4,6-tripyridyl-*S*-trizine (TPTZ), 6-hydroxy-2,5,7,8-tetramethylchromane-2-carboxylic acid (Trolox), gallic acid, BHT, BHA, and vitamin C (V_C_) were purchased from Sigma-Aldrich Chemical Co. (St. Louis, MO, USA). HPD300 macroporous resin was purchased from Cangzhou Bon Adsorber Technology Co., Ltd. (Hebei, China), the surface area 800–870 m^2^/g, the average pore diameter 50–55 nm, the moisture content 75.52%. Prior to the experiments, weighed amounts of resins were soaked in ethanol and subsequently washed by deionized water thoroughly. Then the resins were pretreated by 1 M hydrochloric acid and 1 M sodium hydroxide solution successively to remove the monomers and porogenic agents trapped inside the pores during the synthesis process and washed by deionized water until neutral. All other unlabelled chemicals were of analytical grade and were obtained from Beijing Chemical Reagents Co. (Beijing, China). Reverse osmosis Milli-Q water (Millipore, Bedford, MA, USA) was used for all solutions and dilutions.

### 3.3. Preparation of the Crude Extract and Fractionation

100 g of the ground dried *E. ciliata* sample (inflorescence, leaf, stem and root separately) was mixed with 2 L of water and submitted for 3 h to hydrodistillation, using a Clevenger-type apparatus. The obtained essential oil was dried over anhydrous sodium sulphate for other purposes. After completion of hydrodistillation, the liquid retentate was decanted, filtered through Whatman No. 2 filter paper (Whatman International Limited, Kent, England) and concentrated in a vacuum evaporator (R206B2, Senco Technology Co. Ltd., Shanghai, China) at 60 °C and then lyophilized in a freeze-dryer (FD-1, Beijing Boyikang Lab Instrument Co. Ltd., Beijing, China) to obtain a crude water extract A. 5 g of A was redissolved in 0.5 L of distilled water. The solution was consecutively portioned in a separatory funnel with the equivalent amount of ethyl acetate. The ethyl acetate fraction was concentrated in a vacuum evaporator at 40 °C and then lyophilized in a freeze-dryer to obtain the ethyl acetate fraction B. The aqueous residue was frozen and lyophilized to obtain the fraction C. 1 g of B and C were redissolved in 1 L of distilled water, respectively, the solution was applied to column chromatography on macroporous resin (2 cm × 20 cm) with an isocratic elution 80% ethanol. The ethanol solution was concentrated in a vacuum evaporator at 60 °C and then lyophilized to obtain the fractions D and E (Figure 1). All the five fractions were stored at 4 °C for subsequent analyses.

### 3.4. Determination of Total Phenolics

The total phenolics were determined by Folin-Ciocalteu method [30] with little modification. In brief, 200 μL of samples (10 mg/mL in water) and 1.0 mL of Folin-Ciocalteu reagent (1:1 with water) were mixed. After 4 min, 1.5 mL of 20% sodium carbonate was added and the volume was accurately adjusted to 25 mL with distilled water. The absorbance of the resulting blue color was measured at 765 nm with a UV-vis spectrophotometer (Shimadzu UV-2550; Shimadzu, Kyoto, Japan) after incubation for 60 min at room temperature. The determination was performed in triplicate. Quantification was done on the basis of the standard curve of gallic acid. Results were expressed in g gallic acid equivalent (GAE)/g extracts.

### 3.5. DPPH free Radical-Scavenging Assay

DPPH radical-scavenging activity was determined by the method of [31] with some modifications. Briefly, a 3.8 mL aliquot of freshly prepared DPPH methanol solution (25 μg/mL) was added to 0.2 mL sample solution at different concentrations. The mixture was shaken vigorously and allowed to stand for 30 min in the dark. Then the absorbance was measured at 517 nm in a spectrophotometer. The procedure was performed for different concentrations of each extract. Scavenging activity (SC%) of free radical DPPH in percent (%) was calculated in following way: 


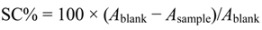
(1)

where *A*_blank_ is the absorbance of the control reaction (containing all reagents except for the test compound), and *A*_sample_ is the absorbance of the test compound. EC_50_ value (μg/mL) is the effective concentration at which DPPH radicals were scavenged by 50% and was obtained by interpolation from regression analysis from the graph plotting inhibition percentage against extract concentration. BHA, BHT and Vc were used as positive control. Tests were carried out in triplicate.

### 3.6. Measurement of Reducing Power

The reducing power was determined as described by [25], but with slight modifications. Briefly, 1 mL each of extracts at different concentrations was mixed with 2.5 mL of 0.2 mol/L phosphate buffer (pH 6.6) and 2.5 mL of potassium ferricyanide (1%). The mixture was then incubated at 50 °C for 20 min. Then, 2.5 mL of 10% trichloroacetic acid (TCA) was added, and the mixture was then centrifuged at 3,000 g for 10 min. The upper layer (2.5 mL) was mixed with 2.5 mL distilled water and 0.5 mL of 0.1% ferric chloride, and the absorbance was measured at 700 nm in a spectrophotometer. Increased absorbance of the reaction mixture indicated higher reducing power. BHA, BHT and Vc were used as positive control. All determinations were performed in triplicate.

### 3.7. Total Antioxidant Capacity by FRAP Assay

The FRAP assay was carried out according to the procedure of [32] with slight modifications. Briefly, the FRAP reagent was prepared from 300 mM sodium acetate buffer (pH 3.6), 10 mM TPTZ solution in 40 mM HCl and 20 mM FeCl_3_ solution in proportions of 10:1:1 (v/v), respectively. The FRAP reagent was prepared fresh daily and was warmed to 37 °C in a water bath prior to use. A 150 μL of sample solution of different concentrations was added to 2.85 mL of FRAP reagent and then standed for 30 min in the dark. The absorbance of the reaction extract solution was recorded at 593 nm. The standard curve was constructed using Trolox solution (37.5–600 μM). In the FRAP assay, the antioxidant efficiency of the antioxidant under the test was calculated with reference to the reaction signal given by Trolox solution of known concentration. The results were corrected for dilution and expressed in μmol Trolox/L. BHA, BHT and Vc were used as positive controls. The assay was carried out in triplicate.

### 3.8. Statistical Analysis

All data were given as means ± SD. One-way analysis of variance (ANOVA) and Duncan’s multiple range test were carried out to determine significant differences (*p* < 0.05) between the means by SPSS (version 10.1).

## 4. Conclusions

This study was designed to evaluate the antioxidant and free radical scavenging activities of extract and fractions from *E. ciliata* using three *in vitro* antioxidant models. Fraction root E, showed the highest total phenolic contents, and followed by fraction root B, fraction stem E and fraction inflorescence E in turn. Meanwhile, fraction root E exhibited higher antioxidant and free-radical scavenging activities, which are stronger than the positive control BHT, BHA and V_C_.

The two enrichment methods of phenolic components: ethyl acetate-water liquid-liquid extraction and macroporous resin adsorption-desorption, were adopted in this study. The macroporous resin technology was effective for enrichment of phenolic components in dealing with ethyl acetate extraction residue and ethyl acetate extract under similar operating parameters.

This study indicated that the extracts from *E. ciliata* have great potential to prevent diseases caused by the overproduction of radicals. The present investigation may provide the evidence required for the utilization of *E. ciliata* as a kind of herbal drug as well as the possibility of using *E. ciliata* as a source of low-cost natural antioxidants. However, further work is still needed to identify and characterise the inherent phytocompounds from relational fractions and to investigate the antioxidant efficacy of *E. ciliata in vivo*.
